# GSK3β Regulates Milk Synthesis in and Proliferation of Dairy Cow Mammary Epithelial Cells via the mTOR/S6K1 Signaling Pathway

**DOI:** 10.3390/molecules19079435

**Published:** 2014-07-03

**Authors:** Xia Zhang, Feng Zhao, Yu Si, Yuling Huang, Cuiping Yu, Chaochao Luo, Na Zhang, Qingzhang Li, Xuejun Gao

**Affiliations:** Key Laboratory of Dairy Science of Ministry of Education, Northeast Agricultural University, Harbin 150030, China; E-Mails: zx291765746@163.com (X.Z.); erjinzhi@126.com (F.Z.); siyu19880329@163.com (Y.S.); hyllw335474663@163.com (Y.H.); yuguiyan@126.com (C.Y.); luochaochao839505@163.com (C.L.); nazhang1981@126.com (N.Z.); qingzhangli2004@163.com (Q.L.)

**Keywords:** glycogen synthase kinase 3β, mTOR, dairy cow mammary epithelial cells, milk synthesis, cell proliferation

## Abstract

Glycogen synthase kinase 3 (GSK3) is a serine/threonine kinase, whose activity is inhibited by AKT phosphorylation. This inhibitory phosphorylation of GSK3β can in turn play a regulatory role through phosphorylation of several proteins (such as mTOR, elF2B) to promote protein synthesis. mTOR is a key regulator in protein synthesis and cell proliferation, and recent studies have shown that both GSK3β and mTORC1 can regulate SREBP1 to promote fat synthesis. Thus far, however, the cross talk between GSK3β and the mTOR pathway in the regulation of milk synthesis and associated cell proliferation is not well understood. In this study the interrelationship between GSK3β and the mTOR/S6K1 signaling pathway leading to milk synthesis and proliferation of dairy cow mammary epithelial cells (DCMECs) was analyzed using techniques including GSK3β overexpression by transfection, GSK3β inhibition, mTOR inhibition and methionine stimulation. The analyses revealed that GSK3β represses the mTOR/S6K1 pathway leading to milk synthesis and cell proliferation of DCMECs, whereas GSK3β phosphorylation enhances this pathway. Conversely, the activated mTOR/S6K1 signaling pathway downregulates GSK3β expression but enhances GSK3β phosphorylation to increase milk synthesis and cell proliferation, whereas inhibition of mTOR leads to upregulation of GSK3β and repression of GSK3β phosphorylation, which in turn decreases milk synthesis, and cell proliferation. These findings indicate that GSK3β and phosphorylated GSK3β regulate milk synthesis and proliferation of DCMECs via the mTOR/S6K1 signaling pathway. These findings provide new insight into the mechanisms of milk synthesis.

## 1. Introduction

Glycogen synthase kinase 3 (GSK3) is a serine/threonine kinase which exists as α and β isoforms. The two isoforms of GSK3 have strongly conserved kinase domains, but differ greatly at the C-terminus. GSK3β plays an important physiological role in the regulation of a wide range of cellular functions including differentiation, growth, apoptosis, and cell response to stimuli [1,2,3]; and GSK3β inactivation or depletion has been shown to promote β-cell proliferation [4]. GSK3β phosphorylates a variety of substrates including glycogen synthase and other metabolic enzymes, transcription factors, and the translation initiation factors [5]. GSK3β is essential for the degradation of CyclinD1, which is an important regulator of G1/S phase cell cycle transition [6]; and in HepG2 and HeLa cells, AKT is known to inactivate GSK3β [7]. GSK3β is furthermore known to induce cell cycle arrest in a CyclinD1-dependent manner in human cells. GSK3β also plays a role in lipid synthesis: GSK3β inactivation can prevent the degradation of the transcription factor SREBP1 [8,9], which is considered a global regulator of lipid metabolism [10,11]. GSK3β can thus promote lipid synthesis.

Lithium chloride (LiCl), a known inhibitor of GSK3β [12], has been used to demonstrate the direct inhibitory effect of lithium on the activity of GSK3 and the subsequent effects on cell signaling and development both *in vitro* and in cells [13]. In humans and rodents, LiCl is known to regulate GSK3β not only directly but also via more complex networks affecting more than one molecular target simultaneously [14].

The mTOR/S6K1 signaling pathway regulates protein and lipid biosynthesis, both of which are processes required for cell growth [15,16]. Amino acids are known to be anabolic factors which induce protein gain by stimulating protein synthesis [17], and the amino acid L-methionine is an essential amino acid that plays fundamental roles in protein synthesis in dairy cow mammary epithelial cells (DCMECs) [18]. One of the conserved roles of mTORC1 is protein synthesis, and in cancer cells, GSK3β may lead to the inactivation of mTORC1 [19]. Moreover, many recent studies have indicated that the inhibition of GSK3β is required for mTOR activation in mice and humans [20,21,22]. Rapamycin, an inhibitor of mTOR, can inhibit cell growth and proliferation [23]. GSK3β acts as a mediator of rapamycin-induced growth inhibition: rapamycin treatment increases the activity of GSK3β and downregulates cyclin D1 levels in a GSK3β-dependent fashion in human breast cancer cell lines [12]. Although there are some reports on the role of GSK3β in milk synthesis, the functional role and details of the mechanism of this role in DCMECs remain largely unknown. The aim of this study was to investigate whether GSK3β could regulate milk synthesis and cell proliferation of DCMECs via the mTOR/S6K1 pathway.

## 2. Results and Discussion

### 2.1. Overexpression of GSK3β Suppresses GSK3β Phosphorylation and the mTOR/S6K1 Signaling Pathway Leading to Milk Synthesis, and Proliferation of DCMECs

**Figure 1:**
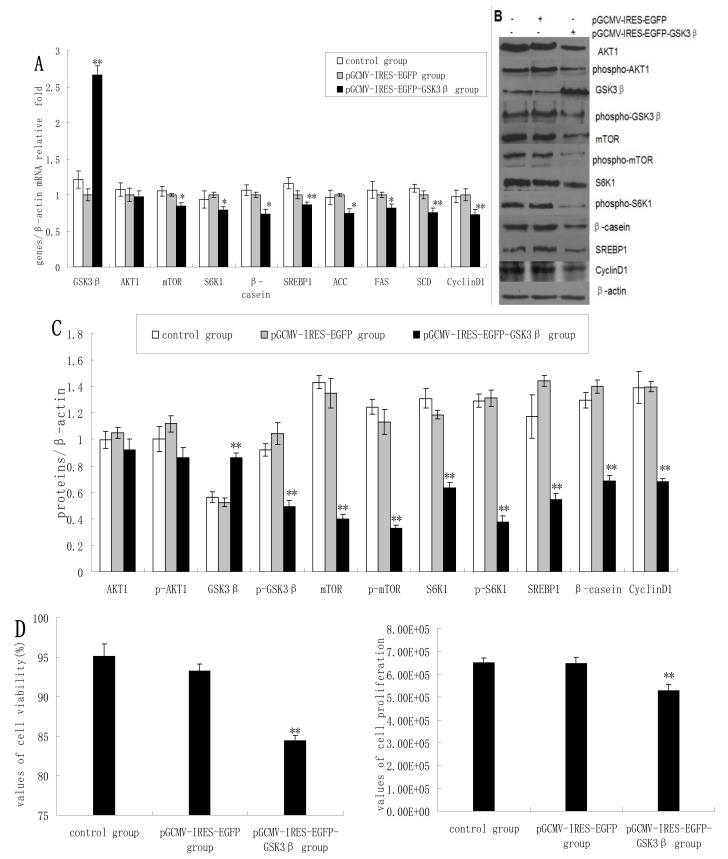

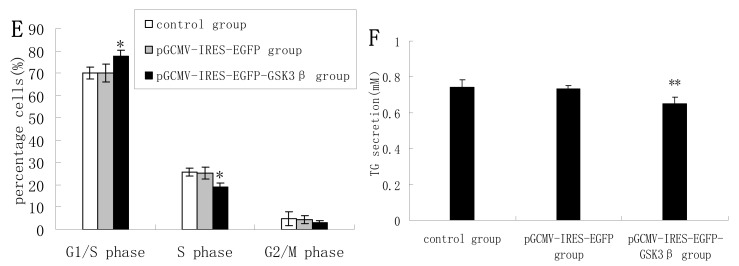
The effects of GSK3β overexpression on gene expression, protein expression, cell proliferation, cell viability, cell cycle, and triglyceride (TG) secretion in DCMECs. Three groups of cells were assessed: untransfected cells, pGCMV-IRES-EGFP empty vector-transfected cells, and pGCMV-IRES-EGFP-GSK3β-transfected cells. Relative mRNA levels (mRNA levels/β-actin) of indicated genes were determined by qRT-PCR after overexpression of GSK3β (**A**) and protein levels were determined by western blotting (**B**) and quantified (proteins/β-actin relative fold) by gray scale scan (**C**). Cell viability and cell proliferation of DCMECs were assessed using a cell counter (**D**) and cell cycle analysis was carried out using flow cytometry (**E**). TG content in DCMEC culture supernatants was quantified (**F**). All values represent means ± SE (*n* = 3 biological replicates). * *p* < 0.05, ** *p* < 0.01 compared with pGCMV-IRES-EGFP empty vector cells.

As expected, the overexpression of GSK3β in DCMECs yielded significantly increased mRNA levels of GSK3β. GSK3β overexpression also resulted in significantly reduced expression of mTOR, S6K1, and β-casein in terms of mRNA levels compared with control cells (empty vector transfectants). In contrast, AKT1 expression was only slightly reduced (Figure 1A) As determined by western blotting analysis, GSK3β overexpression yielded significantly increased GSK3β protein levels, whereas the expression of p-GSK3β, mTOR, p-mTOR, S6K1, p-S6K1, and β-casein was significantly decreased (Figure 1B,C). Beta-casein is the most abundant in milk proteins, and its level can represent the capacity of cells to synthetize milk protein. Cyclin D1 expression was significantly decreased at both mRNA and protein levels following GSK3β overexpression (Figure 1A–C). Overexpression of GSK3β in DCMECs signiﬁcantly inhibited cell growth and viability (Figure 1D). The percentage of cells at G1 phase was increased following GSK3β overexpression, whereas the proportion of cells at S phase was decreased (Figure 1E), indicating that GSK3β activity induced G1 cell cycle arrest in DCMECs. Overexpression of GSK3β in DCMECs downregulated SREBP1, FAS, ACC, and SCD mRNA levels (Figure 1A) and reduced the protein level of SREBP1 (Figure 1B,C) Overexpression of GSK3β in DCMECs significantly decreased the TG content in the culture supernatant of the cells (Figure 1F). The above findings indicate that GSK3β may negatively regulate GSK3β phosphorylation and the mTOR/S6K1 signaling pathway, and may repress milk synthesis and cell proliferation of DCMECs. Numerous reports have confirmed that PI-3-K-Akt-mTOR signaling pathway is responsible for cell proliferation and protein and fat sunthesis in various kinds of cells, including mammary epithelial cells. In other experiment in our laboratory (data not shown), we observed the effect of AKT1 inhibition following siRNA knockdown on mTOR/S6K1 signaling pathway and found AKT1 as a positive regulator on mTOR/S6K1 signaling leading to cell proliferation and milk synthesis. Since AKT1 expression was not significantly reduced by GSK3β overexpression, the observed effects may occur independently of AKT1.

### 2.2. GSK3β Inhibition Enhances GSK3β Phosphorylation and the mTOR/S6K1 Signaling Pathway Leading to Milk Synthesis, and Increases the Proliferation of DCMECs

**Figure 2:**
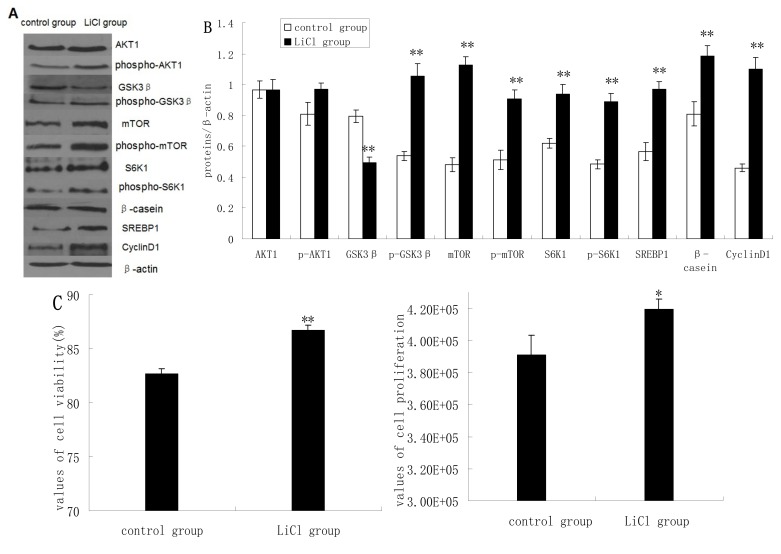

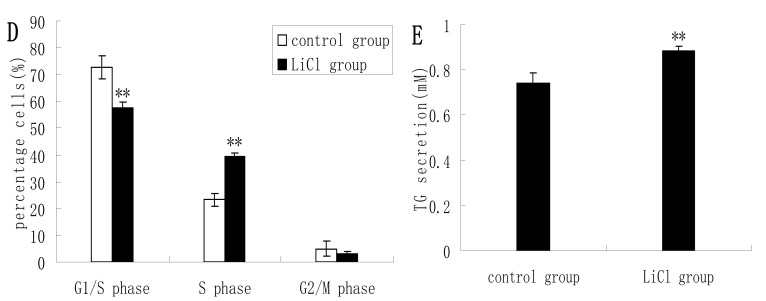
Protein expression, cell proliferation, cell viability, cell cycle, and triglyceride (TG) secretion was assessed in DCMECs treated with LiCl (20 mM) for 24 h and in untreated control cells. Protein expression levels were assessed by western blotting analysis (**A**) and quantified (proteins/β-actin relative fold) by gray scale scan (**B**). Cell viability and cell proliferation of DCMECs were assessed using a cell counter (**C**) and cell cycle analysis was carried out using flow cytometry (**D**). TG content in the culture supernatant of DCMECs was measured (**E**). All values represent means ± SE (*n* = 3 biological replicates). * *p* < 0.05, ** *p* < 0.01 compared with untreated control cells.

GSK3β protein expression was significantly inhibited in cells treated with LiCl (a selective inhibitor of GSK3β), whereas the protein expression levels of p-GSK3β, mTOR, p-mTOR, S6K1, p-S6K1, CyclinD1, SREBP1, and β-casein quantified by western blotting analysis were increased significantly in LiCl-treated cells compared to untreated cells. LiCl treatment yielded only slightly increased expression of AKT1 and p-AKT1 (Figure 2A,B). LiCl treatment enhanced the viability and proliferation of DCMECs (Figure 2C), and in LiCl-treated cells the proportions of cells at G1 phase and S phase were significantly decreased and increased, respectively, compared with untreated cells (Figure 2D). LiCl treatment also led to significantly increased TG secretion in the culture supernatant of DCMECs (Figure 2E). These results indicate that GSK3β inhibition enhances GSK3β phosphorylation and the mTOR/S6K1 signaling pathway leading to milk synthesis and increases proliferation of DCMECs.

### 2.3. Inhibition of the mTOR/S6K1 Signaling Pathway by Rapamycin Blocks the Effects of LiCl on GSK3β

**Figure 3:**
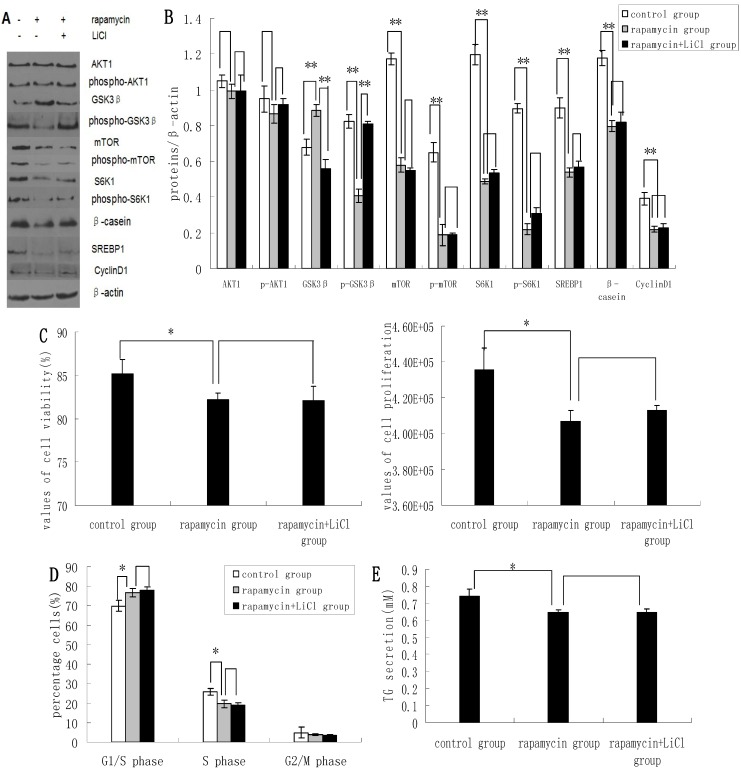
The effects of rapamycin treatment of DCMECs on protein expression, cell proliferation, cell viability, cell cycle, and triglyceride (TG) secretion in the presence and absence of LiCl. Three groups of cells were assessed: a control group (untreated cells), DCMECs treated with rapamycin (1 nM) for 24 h, and DCMECs treated with rapamycin (1 nM) and LiCl (20 mM) for 24 h. Protein expression levels were assessed by western blotting analysis (**A**) and quantified (proteins/β-actin relative fold) by gray scale scan (**B**). Cell viability and cell proliferation of DCMECs were assessed using a cell counter (**C**) and cell cycle analysis was carried out using flow cytometry (**D**). The TG content in the culture supernatants of DCMECs was measured (**E**). All values represent means ± SE (*n* = 3 biological replicates). * *p* < 0.05 and ** *p* < 0.01 indicate significant differences between values across two groups as indicated.

Rapamycin was found to downregulate the expression of p-GSK3β, mTOR, p-mTOR, S6K1, p-S6K1, CyclinD1, SREBP1, and β-casein; and upregulate the expression of GSK3β. Rapamycin treatment had no apparent effect on the expression levels of AKT1 and p-AKT1 (Figure 3A,B). Rapamycin reduced the viability and proliferation of DCMECs (Figure 3C). Compared with untreated control cells, the proportions of rapamycin-treated cells at G1 phase and S1 phase were increased and decreased, respectively (Figure 3D), indicating that rapamycin may block cell cycle progression in DCMECs. Compared with cells treated with both rapamycin and LiCl, rapamycin treatment of DCMECs resulted in an inhibition of the regulatory effects of LiCl on mTOR, p-mTOR, S6K1, p-S6K1, CyclinD1, SREBP1, and β-casein protein expression (Figure 3A,B); on the viability and proliferation of DCMECs (Figure 3C); on cell cycle progression (Figure 3D); and on TG secretion (Figure 3E). On the one hand these findings reveal that inhibition of the mTOR/S6K1 signaling pathway upregulates GSK3β and downregulates GSK3β phosphorylation to inhibit milk synthesis and proliferation of DCMECs; while on the other hand the data suggest that GSK3β may regulate milk synthesis and cell proliferation via the mTOR/S6K1 signaling pathway.

### 2.4. Methionine Activates the mTOR/S6K1 Signaling Pathway and Reverses the Blocking Effect of Rapamycin on GSK3β Phosphorylation by LiCl

**Figure 4:**
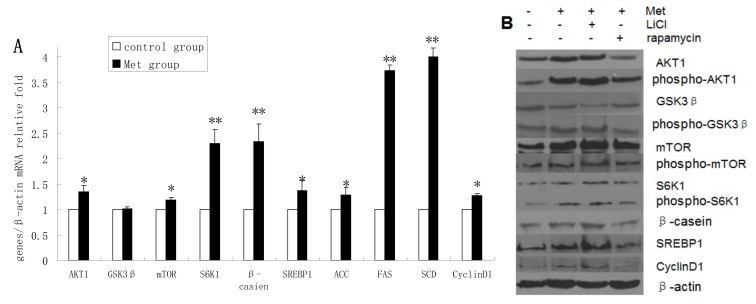

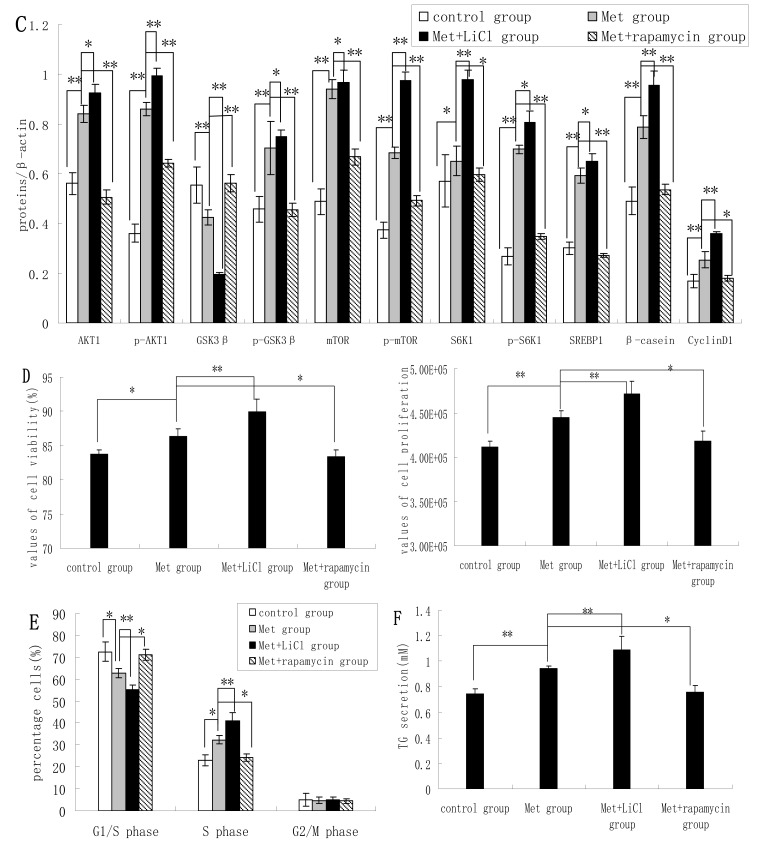
Assessments of gene expression, protein expression, cell viability, cell proliferation, cell cycle, and triglyceride (TG) secretion in four groups of DCMECs: control (untreated) cells, DCMECs treated with Met (0.6 mM) for 24 h, DCMECs treated with Met (0.6 mM) and LiCl (20 mM) for 24 h, and DCMECs treated with Met (0.6 mM) and rapamycin (1 nM) for 24 h. Relative mRNA levels (mRNA levels/β-actin) were determined by qRT-PCR following Met treatment (**A**) and protein levels were assessed by western blot analysis (**B**) and quantified (proteins/β-actin relative fold) by gray scale scan (**C**). Cell viability and cell proliferation of DCMECs were assessed using a cell counter (**D**) and cell cycle analysis was carried out using flow cytometry (**E**). The TG content in the culture supernatants of DCMECs was measured (**F**). All values represent means ± SE (*n* = 3 biological replicates). * *p* < 0.05 and ** *p* < 0.01 indicate significant differences between values across two groups as indicated.

The mRNA levels of AKT1, mTOR, S6K1, β-casein, SREBP1, ACC, FAS, SCD, and cyclin D1 were upregulated in Met-treated cells compared with untreated cells, whereas Met treatment had no obvious effect on GSK3β mRNA expression (Figure 4A). The protein levels of AKT1, p-AKT1, p-GSK3β, mTOR, p-mTOR, S6K1, p-S6K1, β-casein, SREBP1, and cyclin D1 were increased in Met-treated cells compared with untreated cells, whereas GSK3β protein expression was downregulated following Met treatment (Figure 4B,C). Met was found to improve the viability and proliferation of DCMECs (Figure 4D). In Met-treated cells there was a significant decrease in the proportion of cells at G1 phase and an increase at S phase compared with untreated cells (Figure 4E). TG secretion was also enhanced in Met-treated cells (Figure 4F). These results indicate that Met activates the mTOR/S6K1 signaling pathway and enhances GSK3β phosphorylation to upregulate milk synthesis and cell proliferation of DCMECs.

Treatment of DCMECs with both Met and LiCl resulted in further increases in the protein levels of SREBP1, β-casein, p-GSK3β, S6K1, and mTOR; and decreased GSK3β expression. Compared with expression levels in cells treated with Met alone, treatment with Met and rapamycin reduced the expression levels of these proteins, except in the case of GSK3β, the expression of which was increased. This indicates that Met activates the mTOR/S6K1 signaling pathway to enhance GSK3β phosphorylation. The viability and proliferation of DCMECs treated with both LiCl and Met was higher than in cells treated with Met only, while proliferation and viability was lower in cells treated with both rapamycin and Met compared with cells treated with only Met (Figure 4D). Compared with Met-treated cells, the proportion of cells at G1 phase in cells treated with both LiCl and Met was reduced, and the proportion of cells at S phase was increased (Figure 4E). In cells treated with both rapamycin and Met, the proportion of cells at G1 phase was increased compared with cells treated with only Met, and the population of cells at S phase was decreased (Figure 4E). Compared with cells treated with Met only, TG secretion was enhanced in cells treated with both LiCl and Met, and reduced in cells treated with both rapamycin and Met (Figure 4F). These results provide further evidence for the hypothesis that GSK3β phosphorylation is regulated by the mTOR/S6K1 signaling pathway to control milk synthesis and cell proliferation proliferation of DCMECs; and that, on the other hand, the regulatory effects of GSK3β on milk synthesis and cell proliferation are associated with the mTOR/S6K1 signaling pathway.

### 2.5. Discussion

GSK3β is involved in modulating a variety of functions including growth, metabolism, and protein and lipid synthesis [23,24,25]. GSK3β is an inhibitor of the mTOR/S6K1 signaling pathway; however, mTOR can also inhibit GSK3β activity. This work is the first report addressing the question of whether or not GSK3β and its different molecular forms can regulate milk synthesis and proliferation of DCMECs via the mTOR/S6K1 signaling pathway. Our experimental results indicate that phosphor-GSK3 beta might have the kinase activity on mTOR/S6K1 pathway, whereas unphosphorylated GSK3β might have the phosphatase activity. Since we used the antibody to GSK3 beta (phospho S9) to determine the ectopic expression of GSK-3β to inhibit pGSK3β, the inhibition is at least at ser9 and reflects a decrease in the inhibition of GSK3β– hence activation. This speculation needs further study.

The regulation of protein biosynthesis in all tissues of mammals appears to be under the control of the mTOR pathway. Recent work in rodents and ruminants has highlighted a role for mTOR in regulating milk protein synthesis [26]. The mTOR-S6K1 axis plays important roles in cell growth, proliferation, and cell differentiation by regulating protein synthesis and lipid metabolism [27]. To assess the effects of GSK3β inhibition on milk synthesis, we measured changes in the protein expression levels of AKT1, GSK3β, mTOR, S6K1, and β-casein. GSK3β inhibition with LiCl resulted in increased expression of β-casein, as well as increased protein levels of the phosphorylated forms of GSK3β, mTOR, and S6K1. Previous reports have highlighted that insulin activates the PI3K/AKT pathway and inhibits GSK3β activity giving rise to its inactivation and activation of mTOR pathways [28]. In the current study LiCl is shown to increase the phosphorylation of GSK3β and S6K1 while, in contrast, LiCl was found to have no significant effect on AKT1 phosphorylation. The same observations have also been made in HEK293, MEF, and LEF cells [15]. Overexpression of GSK3β was found to repress the phosphorylation of GSK3β, mTOR, and S6K1; and the expression of β-casein, while having no significant effect on AKT1. In bovine luteal cells, the GSK3β inhibitor LiCl has been shown to stimulate the phosphorylation of S6K1 [29]. Studies over the past decade have uncovered a number of additional S6K1 substrates besides rps6 (ribosomal protein S6), revealing that S6K1 mediates many aspects of cell physiology, including cell proliferation, protein and fat synthesis [30,31]. Our findings reveal that GSK3β can reduce milk protein synthesis in DCMECs by inhibition of the mTOR/S6K1 signaling pathway, possibly by exerting phosphatase activity. The mechanism of milk synthesis regulation by GSK3β, however, requires further investigation.

Lipid synthesis involves the *de novo* synthesis of fatty acids (FA) and preformed FA into triglycerides. SREBP1 translocates to the nucleus where it activates lipogenic genes including FAS, ACC, and SCD by binding to the SREBP1 response element of target genes [32]. To assess the effects of GSK3β on milk fat synthesis, the expression of genes associated with fat synthesis and triglyceride secretion was measured. The protein expression of SREBP1 was increased in LiCl-treated cells. Triglyceride secretion was also increased. Conversely, overexpression of GSK3β resulted in downregulated ACC, FAS, SCD, and SREBP1 expression at mRNA and protein levels, and led to decreased of triglyceride secretion. SREBP1 activation and lipogenesis require mTOR [33,34], and thus our findings indicate that GSK3β inhibition may upregulate the expression of SREBP1 to increase milk fat synthesis in DCMECs via the mTOR/S6K1 signaling pathway.

Proliferation of DCMECs is a determining factor for milk production [35]. Cell viability and proliferation of DCMECs were significantly enhanced by LiCl treatment but reduced following overexpression of GSK3β. Inhibition of GSK3β promoted G1/S transition in DCMECs whereas overexpression of GSK3β resulted in repressed G1/S transition. Overexpression of GSK3β, a cyclin D1 protein kinase, led to significantly reduced cyclin D1 protein expression, while LiCl treatment resulted in an apparent increase in cyclin D1 expression. Cyclin D1 is an important regulator of G1/S phase cell cycle transition and its proteolysis is thought to be mediated by GSK3β-induced degradation [12,36]. In our study, the viability, proliferation, and G1/S phase cell cycle transition of DCMECs were extremely reduced by rapamycin treatment. To analyze the impact of the mTOR pathway on GSK3β, cells were treated with rapamycin in the presence and absence of LiCl. No significant differences were observed between the two sets of conditions, indicating that the LiCl-mediated promotion of proliferation in DCMECs is blocked by rapamycin. We thus propose a possible mechanism in which GSK3β affects cell cycle progression via the mTOR/S6K1 signaling pathway leading to cyclin D1 degradation in DCMECs.

In our investigation rapamycin treatment was found to upregulate the expression of GSK3β in DCMECs, and to reduce the expression of p-GSK3β, mTOR, p-mTOR, S6K1, p-S6K1, and β-casein, while the treatment had no apparent effect on the expression levels of AKT1 and p-AKT1 in DCMECs. Previously, George *et al.* showed that mTOR inhibition by rapamycin leads to reduced expression of mTOR pathway-related proteins in cultured podocytes [37]. Rapamycin-induced inhibition of mTOR has furthermore been shown to result in reduced phosphorylation of GSK3β [38]. We found SREBP1 expression to be significantly reduced following rapamycin treatment. Porstmann *et al.* [23] demonstrated that events involved in lipogenesis, including nuclear accumulation of SREBP1 and induction of SREBP1 target genes, are inhibited by rapamycin treatment. In our study, rapamycin blocked the stimulatory effects of LiCl on GSK3β phosphorylation and repressed β-casein expression, SREBP1 expression, TG secretion, and cell cycle progression. These observations further support our hypothesis that GSK3β phosphorylation increases milk synthesis and DCMEC proliferation via the mTOR/S6K1 signaling pathway.

**Figure 5:**
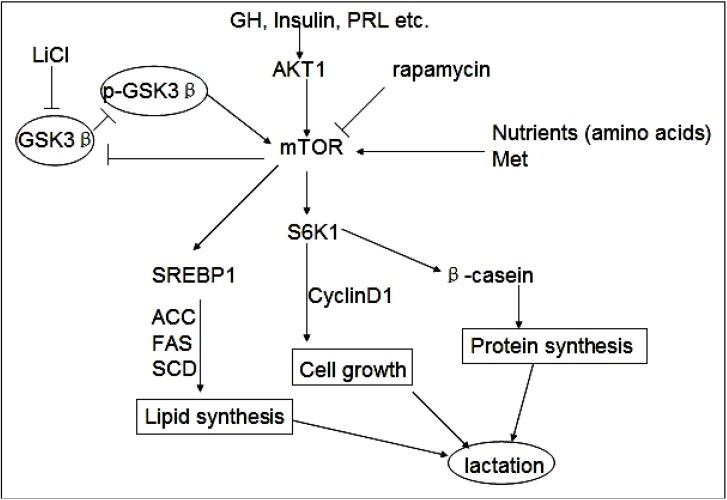
GSK3β and phospho-GSK3β regulate milk synthesis and DCMEC proliferation via the mTOR/S6K1 signaling pathway. GSK3β inhibits the mTOR/S6K1 signaling pathway involved in milk protein and fat synthesis, as well as the proliferation of DCMECs. The direct inhibition of GSK3β by LiCl can down-regulate GSK3β expression and promote GSK3β phosphorylation to activate the mTOR/S6K1 signaling pathway. GSK3β downregulates cyclin D1 and inhibits cell growth via the mTOR/S6K1 pathway. Upregulated expression of lipogenic genes including ACC, SCD, and FAS through activation of SREBP1 is required for efficient induction of lipogenesis by the mTOR/S6K1 pathway. Rapamycin represses the mTOR/S6K1 pathway involved in milk synthesis and cell proliferation and reverses the effect of p-GSK3β.

Met plays fundamental roles in milk protein synthesis and a number of other biochemical processes including cell proliferation [38,39]. Parisi *et al.* found that amino acids increase the stability of Myc proteins by GSK3β inhibition in Drosophila S2 cells [40]. Using a similar biochemical approach, we analyzed the effects of Met on milk synthesis and cell proliferation as in investigation into how mTOR signaling is linked to the mechanisms by which inactivation of GSK3β promotes milk synthesis and DCMEC proliferation. In our laboratory, we have confirmed that Met is the most effective amino acid to enhance mTOR pathway leading to cell proliferation and milk synthesis. Other amino acids such as Leu, Phe, Thr, Trp also have the effects, but the effects are less than Met and gradually become weak (data not shown). In these experiments we demonstrated that Met enhances the mTOR/S6K1 signaling pathway, increases the viability and proliferation of DCMECs, and increases the expression of cyclin D1, SREBP1, β-casein, as well as TG secretion. These increases were also accompanied by an increase in GSK3β phosphorylation. Met treatment resulted in increased SREBP1, ACC, FAS, and SCD expression at an mRNA level or protein level, as well as in increased TG secretion, highlighting the fact that this amino acid not only promotes protein synthesis, but also induces lipid synthesis. To ascertain whether Met contributes to GSK3β activity in milk protein and fat synthesis, DCMECs were stimulated with Met in the presence and absence of either LiCl or rapamycin. It was then assessed whether GSK3β could regulate milk accumulation and cell proliferation under this treatment condition. Our findings indicated that Met reverses the cutoff effects of rapamycin on milk synthesis and cell proliferation. Conversely, the LiCl effect is reversed by Met in the presence of rapamycin. These observations further support our hypothesis that the functions of GSK3β and its phosphorylated form are associated with the mTOR/S6K1 signaling pathway leading to milk synthesis and DCMEC proliferation (Figure 5).

## 3. Experimental Section

### 3.1. Instruments and Materials

DT CASY cell counter (CASY, Schärfe System GmbH, Reutlingen, Germany); FACS Calibur flow cytometer (Becton Dickinson, Trenton, NJ, USA); ABI PRISM 7300 RT-PCR System (Applied Biosystems, Foster City, CA, USA).

Dulbecco’s Modified Eagle’s medium-F12 (DF-12; Gibco, Carlsbad City, CA, USA); methionine (Met; Sigma, St. Louis, MO, USA); lithium chloride (LiCl; Abcam, Cambridge, England); rapamycin (Cell Signaling, Danfoss, MA, USA); rabbit polyclonal antibodies to GSK3β (Abcam); goat polyclonal antibodies to p-GSK3β (phospho S9) and AKT1 (Santa Cruz, Palo Alto, CA, USA); p-AKT1, mTOR, p-mTOR, and p-S6K1 (Cell Signaling, Danfoss, MA, USA); S6K1, SREBP1, cyclin D1, and β-actin (Santa Cruz, Palo Alto, CA, USA); and β-casein (Abbiotec, San Diego, CA, USA); triglyceride (TG) GPO-POD assay kit (Applygen Tech Inc., Beijing, China).

### 3.2. Cell Preparation and Treatments

Primary DCMECs were cultured and purified using previously described protocols [18,41]. Briefly, purified DCMECs were cultured in DF-12 medium. For experimental assays, DCMECs in the logarithmic growth phase were plated at 3 × 10^4^ cells cm^−2^.

### 3.3. Inhibition of GSK3β and mTOR

DCMECs were treated with LiCl (20 mM), a selective inhibitor of GSK3β, or rapamycin (1 nM), a selective inhibitor of mTOR [9], and incubated at 37 °C, 5% CO_2_ for 24 h before collection for further experiments.

### 3.4. pGCMV-IRES-EGFP-GSK3β Vector Construction and Transfection

The full-length GSK3β gene was amplified by PCR using primers designed with specific restriction enzyme sites: 5'-GAAGATCTAAAGAGTGATCATGTCAGGGCG-3' (*Bgl* II), forward; and 5'-ACGCGTCGACCTGGCTGCTCGGGACTGTTC-3' (*Sal* I), reverse (NCBI accession number: NM_001101310.1). The resulting PCR fragment was subcloned into a TA cloning vector (Promega, Madison, Wi, USA) and was sequenced. The full-length GSK3β gene was obtained from the cloning vector by digestion with *Bgl* II and *Sal* I after which the gene was subcloned into a pGCMV-IRES-EGFP vector (GenePharma, Shanghai, China). All constructs were subjected to sequencing for verification. DCMECs were transfected with either the pGCMV-IRES-EGFP vector (empty vector control) or the pGCMV-IRES-EGFP-GSK3β vector using LipofectamineTM 2000 (LF2000; Invitrogen, Carlsbad City, CA, USA) according to the manufacturer’s instructions. Cells were incubated at 37 °C, 5% CO_2_ for 24 h, after which they were collected for further experiments.

### 3.5. Treatment of DCMECs with Methionine

DCMECs were treated with Met (0.6 mmol/L) [18], Met and LiCl (20 mM), or Met and rapamycin (1 nM) and incubated at 37 °C, 5% CO_2_ for 24 h after which they were collected for further experiments.

### 3.6. Cell Viability and Proliferation Assessments

Cell viability and proliferation was determined by using a DT CASY cell counter as described in previous reports [37,42].

### 3.7. Cell Cycle Assessment

Cells were washed with cold PBS, trypsinized, and collected by centrifugation, after which they were fixed with 80% cold ethanol at 4 °C overnight. Before analysis, cells were washed, collected and resuspended in PBS containing 50 µg/mL propidium iodide (PI) in 2 µL/mL TritonX-100. Cells were then incubated at room temperature in the dark for at least 30 min. After resuspension in 500 µL PBS, cells were subjected to flow cytometry. The results of flow cytometry assessments were calculated as the proportion (%) of cells in each cell cycle phase.

### 3.8. Detection of Triglyceride Secretion

Cell-free supernatants were assessed for TG secretion using the Triglyceride GPO-POD assay Kit (Applygen Tech Inc.) according to the manufacturer’s instructions.

### 3.9. RNA Extraction and Quantitative Real Time PCR

Total RNA from DCMECs was extracted using Trizol reagent according to the manufacturer’s instructions, after which total RNA (1 μg) was transcribed into cDNA according to the manufacturer’s protocol. After reverse transcription, qRT-PCR was carried out using the comparative CT method. β-actin was used as the reference gene and the oligonucleotide sequences of the forward and reverse primers for the target genes are shown in Table 1.

**Table 1:** Primer sequences for qRT-PCR analysis.

Primer Name	Primer Sequences (5' → 3')	
	Forward Primer	Reverse Primer
β-actin	AAGGACCTCTACGCCAACACG	TTTGCGGTGGACGATGGAG
AKT1	TAAAGAAGGAGGTCATCGTGG	CGGGACAGGTGGAAGAAAA
GSK3β	CCATCCTTATTCCTCCTC	TTGGTCTGTCCACGGTCT
mTOR	ATGCTGTCCCTGGTCCTTATG	GGGTCAGAGAGTGGCCTTCAA
S6K1	AAATGCTGCTTCTCGTCT	GTTCTTCCCAGTTAATATGTCT
β-casein	AACAGCCTCCCACAAAAC	AGCCATAGCCTCCTTCAC
SREBP1	AGTAGCAGCGGTGGAAGT	GCAGCGGCTCTGGATT
ACC	AGACAAACAGGGACCATT	AGGGACTGCCGAAACAT
FAS	CCACGGCTGTCGGTAAT	CGCTCCCACTCATCCTG
SCD	CTGTGGAGTCACCGAACC	TAGCGTGGAACCCTTTT
CyclinD1	CCGTCCATGCGGAAGATC	CAGGAAGCGGTCCAGGTAG

### 3.10. Western Blotting Analysis

Western blotting analysis was performed according to previously described protocols used to detect protein expression of GSK3β, p-GSK3β, mTOR, p-mTOR, S6K1, p-S6K1, β-casein, SREBP1, cyclin D1, and β-actin [37]. Three parallel groups were prepared for each treatment. Gray-scale scanning of western blotting results was analyzed by Glyko Band Scan 5.0 software, β-actin was used as the loading control.

### 3.11. Statistical Analysis

All results are reported as mean ± SE. Statistics and differences between groups were analyzed using t-tests (SPSS 17.0 software). Differences with *p* < 0.05 were considered statistically significant and differences with *p* < 0.01 were considered highly significant. Results of cell cycle assessments by flow cytometry were analyzed using Modfit LT 3.2 software and gray-scale scanning of western blotting results were analyzed using Glyko Band Scan 5.0 software.

## 4. Conclusions

GSK3β represses the mTOR/S6K1 signaling pathway, whereas inhibition of GSK3β leads to GSK3β phosphorylation, which enhances the mTOR/S6K1 signaling pathway leading to milk synthesis and DCMEC proliferation.
